# Comparative Seasonal Respiratory Virus Epidemic Timing in Utah

**DOI:** 10.3390/v12030275

**Published:** 2020-02-29

**Authors:** Zayne Y. Callahan, Trevor K. Smith, Celeste Ingersoll, Rebecca Gardner, E. Kent Korgenski, Chantel D. Sloan

**Affiliations:** 1 Department of Public Health, Brigham Young University, Provo, UT 84602, USA; zayneycallahan@gmail.com (Z.Y.C.); trevorkent1@gmail.com (T.K.S.); 2 Department of Statistics, Brigham Young University, Provo, UT 84602, USA; celesteingersoll3@gmail.com (C.I.); rbecca22@gmail.com (R.G.); 3 Pediatric Clinical Program, Intermountain Health Care, Salt Lake City, UT 84102, USA; Kent.Korgenski@imail.org

**Keywords:** influenza, viral interference, wavelet

## Abstract

Previous studies have found evidence of viral interference between seasonal respiratory viruses. Using laboratory-confirmed data from a Utah-based healthcare provider, Intermountain Health Care, we analyzed the time-specific patterns of respiratory syncytial virus (RSV), influenza A, influenza B, human metapneumovirus, rhinovirus, and enterovirus circulation from 2004 to 2018, using descriptive methods and wavelet analysis (*n* = 89,462) on a local level. The results showed that RSV virus dynamics in Utah were the most consistent of any of the viruses studied, and that the other seasonal viruses were generally in synchrony with RSV, except for enterovirus (which mostly occurs late summer to early fall) and influenza A and B during pandemic years.

## 1. Introduction

Seasonal viruses are responsible for hundreds of thousands of deaths and extensive morbidity in temperate climates each year [1,2]. In the United States, epidemics of respiratory syncytial virus (RSV) and influenza typically begin in the Southeast United States and progress to the Northwest, through the months of October to April [3,4,5]. Much of the seasonal timing and geographic spread between respiratory viruses coincide, resulting in high prevalence of coinfection globally, which may potentially be linked to disease severity [6,7,8].

Competition for host cells during coinfection can result in viral interference in the form of delaying or preventing infection by the secondary virus [9,10]. It is possible that this cellular interference may be detectable on a population level. For example, research suggests that epidemics of RSV, coronavirus, and influenza B can be respectively delayed, intensified, or inhibited if circulation of influenza A begins early (before week one of a given seasonal year) [11]. Furthermore, faster growing seasonal viruses, such as rhinovirus, may reduce the rate of replication of slower growing seasonal viruses, while RSV infection may in turn reduce the risk of rhinovirus coinfection [9,12].

The complex ecological interactions between viruses are difficult to discern using typical measures of correlation, since relationships between epidemics may shift over time, depending on strain severity, host immunity, and climate factors. In this study, we compared epidemic timing and calculated phase differences between seasonal epidemics of RSV, influenza A and B, metapneumovirus, enterovirus, and rhinovirus, using descriptive and wavelet analyses. Wavelet analysis allows for viewing changes in epidemic synchronistic patterns over multiple years [13,14].

## 2. Materials and Methods

### 2.1. GermWatch^®^ Data

We obtained weekly frequency data for six seasonal respiratory viruses (influenza A and B, RSV, metapneumovirus, enterovirus, and rhinovirus), from Intermountain Healthcare’s GermWatch^®^ database, for the 2005–2006 winter season to the 2017–2018 winter season. We did not have data for summer 2018, nor did we have access to data at the individual level. Intermountain Healthcare is the largest health-care provider in the Intermountain West region of the United States and operates a nonprofit system of 22 hospitals and more than 190 clinics. The study was considered exempt by the Institutional Review Board at Brigham Young University.

The principal laboratory methods of virus identification were direct fluorescent antibody (DFA), viral culture, and rapid antigen testing between 2000 and 2007. After 2007, polymerase chain reaction (PCR) methodology became the primary method of identification, but DFA and rapid antigen testing was still available. Due to the aggregated nature of our dataset, it is not possible to differentiate between which tests were used for each data point. However, the more sensitive method (i.e., PCR > DFA > rapid antigen testing) was used in the analysis if different methods were ordered and to exclude duplicate test results.

### 2.2. Analysis

We conducted two separate analyses to compare epidemic timing between viruses over the study period. The first was a descriptive analysis in which epidemic initiation, peak, and termination were identified based on a change point model. Second, we conducted a wavelet analysis to compare epidemic synchrony across seasons. All analyses were conducted in R (v. 3.5.1) [15]. Note that five of the viruses studied occur during the fall and winter seasons, while enterovirus infection typically occurs during late summer in temperate climates.

Wavelet analysis and change point models are complementary methods we implemented to investigate the timing and patterns of epidemics. For our purposes, the strength of change point models over others, such as SIR models or circular statistics, is that they give more precise estimates of when different phases of a past epidemic began and terminated. Wavelet analysis compares epidemic timing in a way that allows for the behavior of the epidemic to change in different years. This would be more difficult if using methods that employ sine waves to estimate epidemic curves. We can therefore make direct comparisons of the historical characteristics of the epidemics (via change point models) and their synchrony with one another (wavelet analysis), using our selected methods. We recognize that there are many other methods that would lend different insights into the epidemic patterns described, but we deemed these two sufficient for the current study.

#### 2.2.1. Descriptive Analysis

For the descriptive analysis, we used a statistical change point model that was recently utilized to analyze RSV circulation throughout the United States [16]. We used this model to determine seasonal patterns in epidemic initiation, peak, and termination for all six viruses previously mentioned. For each year and virus, a base value count was determined as a starting value for the curve. Afterward, slopes of the mean counts were found, starting at base value, and four change points were identified: T1–T4. These data were used to create a line plot depicting the mean frequency count for each virus during each seasonal year.

**Figure 1:**
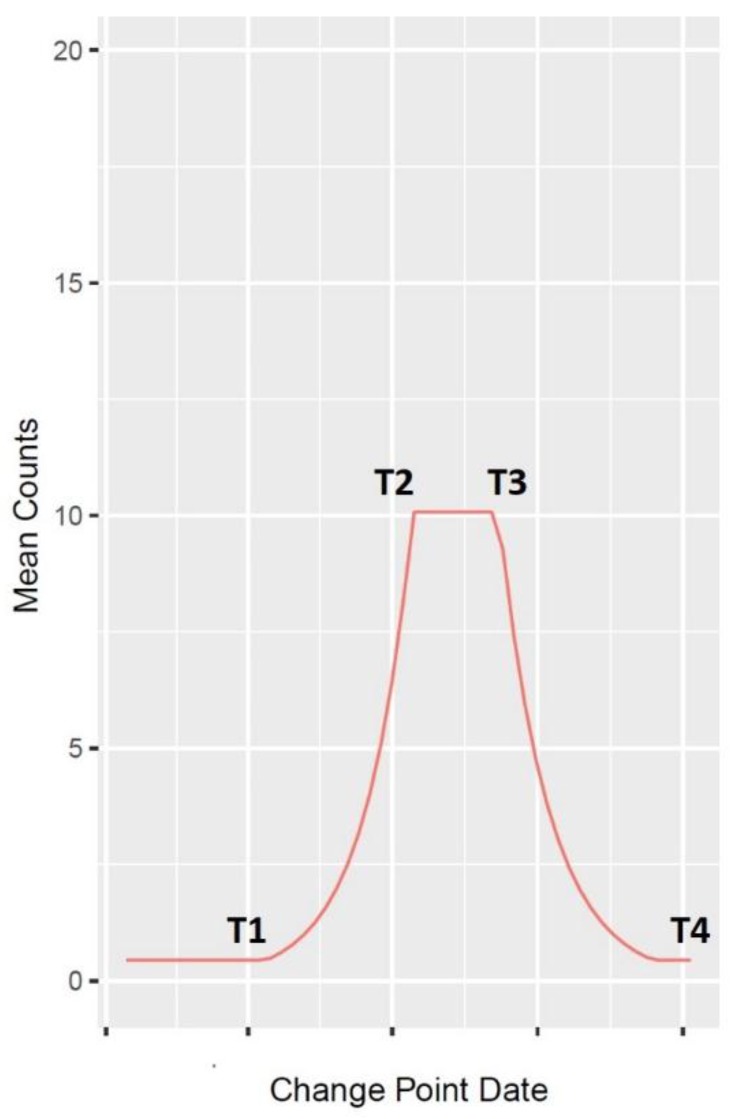
Change point plot description.

A seasonal year is defined as starting on 1 July and ending on 30 June of the following year (e.g., seasonal year 2004 represents 1 July 2003 to 30 June 2004). The line between change points T1 and T2 represents the slope of virus onset to epidemic peak. The line between change points T2 and T3 represents the epidemic peak duration. The line between change points T3 and T4 represents the virus decline to termination (Figure 1).

Data are lacking for metapneumovirus prior to January 2007 and for rhinovirus prior to November 2007. Enterovirus is shown in a separate line plot due to its having a significantly lower mean count than the five other viruses, and it is plotted on a 1 January to 31 December cycle rather than the regular seasonal year.

In order to validate our method, we compared our estimates of peak and epidemic duration of RSV with those reported by the National Respiratory and Enteric Virus Surveillance System (NREVSS) [17]. Denver was the closest NREVSS surveillance site and is generally used to describe trends in the Western Region of the United States. NREVSS indicates onset, peak, and offset dates for RSV trends. Of the 9 seasonal years that NREVSS indicated peak dates, 7 of them fell within our peak durations (T2 and T3).

#### 2.2.2. Wavelet Analysis

To conduct the wavelet analysis, we first normalized the frequency data to have a mean of zero. We then calculated the restructured component of the epidemic curves, using a period window of 32 to 65 weeks for each virus, except for influenza A and B. For the influenza viruses, a longer period of 108 weeks (2 years) was necessary to accurately capture epidemic patterns in pandemic years (2009–2010). A loess smoother was applied to the rhinovirus data, to adjust for increases in laboratory testing in late 2007 and early 2008. The period windows were chosen based on cross-wavelet power levels with RSV (see Figure A4). RSV was selected as the reference due to its very consistent repeating annual pattern.

We plotted the phase angles and calculated the phase difference between each virus. Plots of each step of the analysis are found in Figure A3a–e. In Figure A3e, synchrony between the epidemic curves is determined by the phase difference being zero, while deviation from the zero line indicates the epidemics are out of phase. Wavelet analyses were done by using the R package WaveletComp [18].

Wavelet analysis is nuanced but has many features that are similar to other wave decomposition methods. It has been shown to be a useful tool in comparing infectious disease rates in several studies [13,19]. Its strength is in allowing the timing of the epidemic curves to change from year to year by using a wavelet instead of something more regular such as a sine curve. This allowed for greater flexibility and precision in identifying epidemic trends that may change from year to year.

## 3. Results

### 3.1. Descriptive Analysis

**Figure 2:**
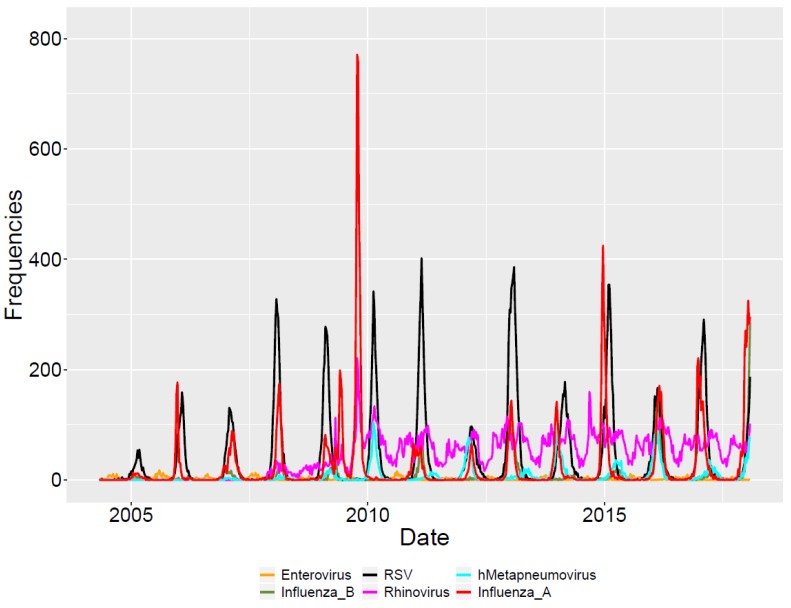
Raw frequency plot.

The dataset contained 28,671 laboratory-confirmed cases of RSV; 18,451 cases of influenza A; 4767 cases of influenza B; 1347 cases of Enterovirus; 4945 cases of metapneumovirus; and 31,281 cases of rhinovirus over 13 years (total = 89,462). (See Figure 2 for raw frequency plot of all viruses. See Table A1 for frequency data of each virus in each seasonal year.)

The highest and lowest frequencies of RSV were in seasonal year 2013 (4002 cases) and 2005 (506 cases), respectively. The majority of RSV cases occurred between January and March, with the most common month being February (see Table A2 for all monthly frequency data). Metapneumovirus rates were highest in seasonal year 2014 (821 cases) and lowest in 2008 (81 cases), with the majority of peak timing usually occurring between January and March, and the most common month being February.

The highest and lowest frequencies of influenza A were in seasonal year 2010 (3693 cases) and 2005 (132 cases), respectively. The earliest and latest timing of the peak of influenza A occurred in 2010 and 2012, respectively. Peak timing for influenza A was less consistent compared to other viral infections, primarily due to 2009 H1N1, but usually occurred between December and January.

**Figure 3:**
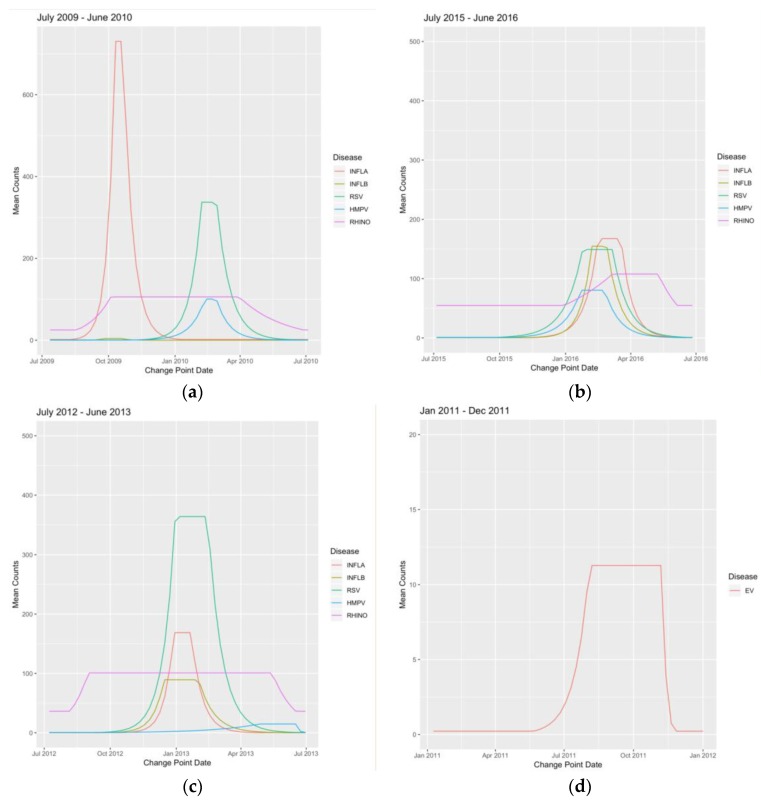
Change point plots: (**a**) influenza A and human Metapneumovirus exhibited their highest mean counts in the 2009–2010 season; (**b**) influenza B exhibited its highest mean count in the 2012–2013 season; (**c**) respiratory syncytial virus (RSV) and rhinovirus exhibited their highest mean counts in the 2012–2013 season; and (**d**) enterovirus (EV) exhibited its highest mean count in 2011.

**Figure A1:**
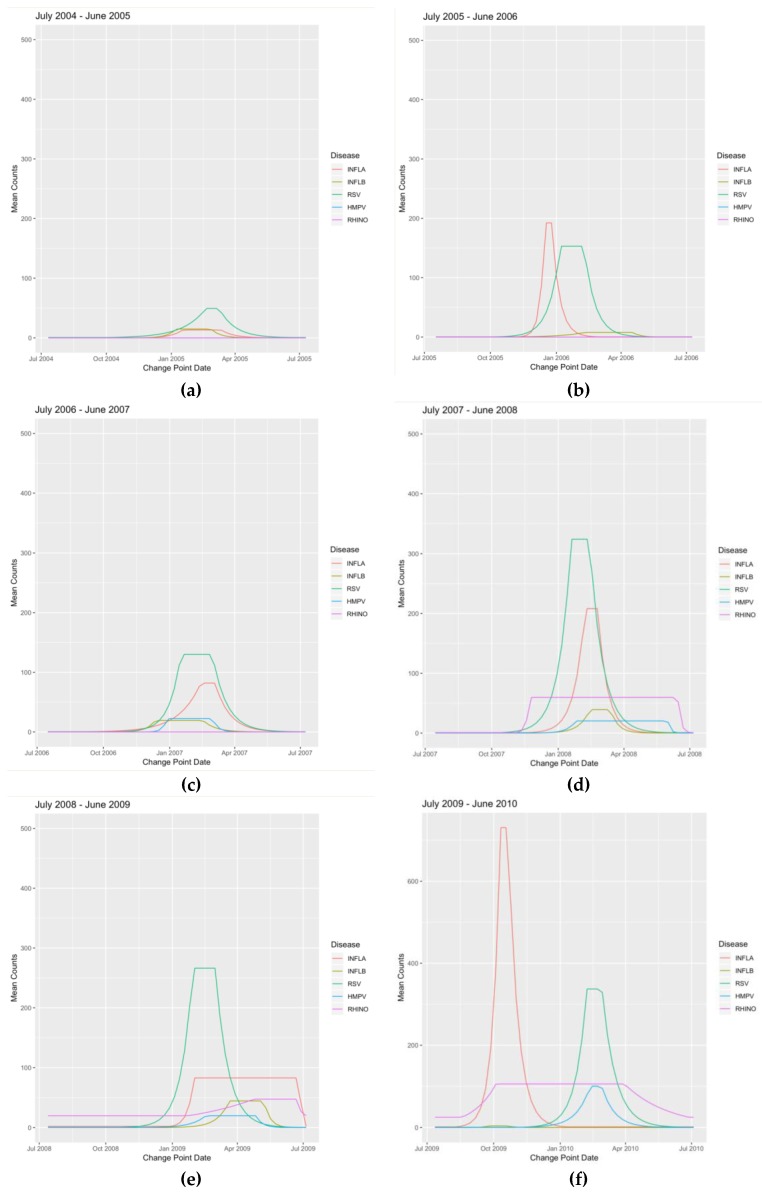

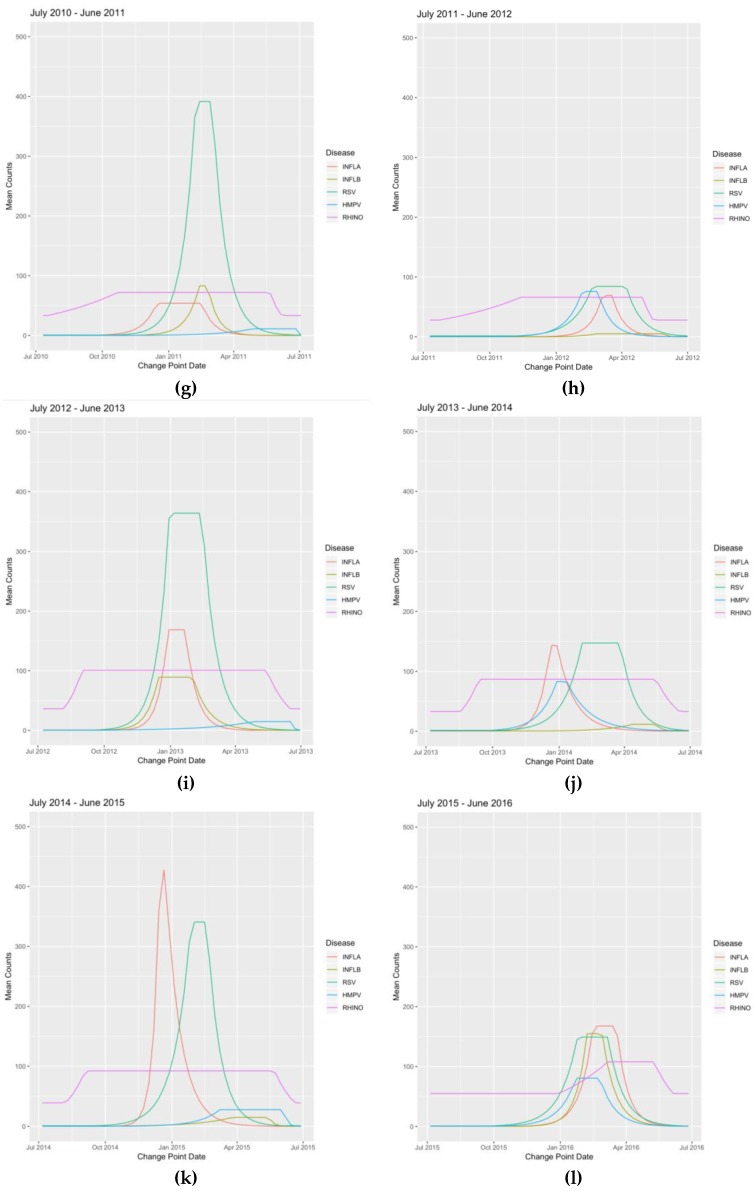

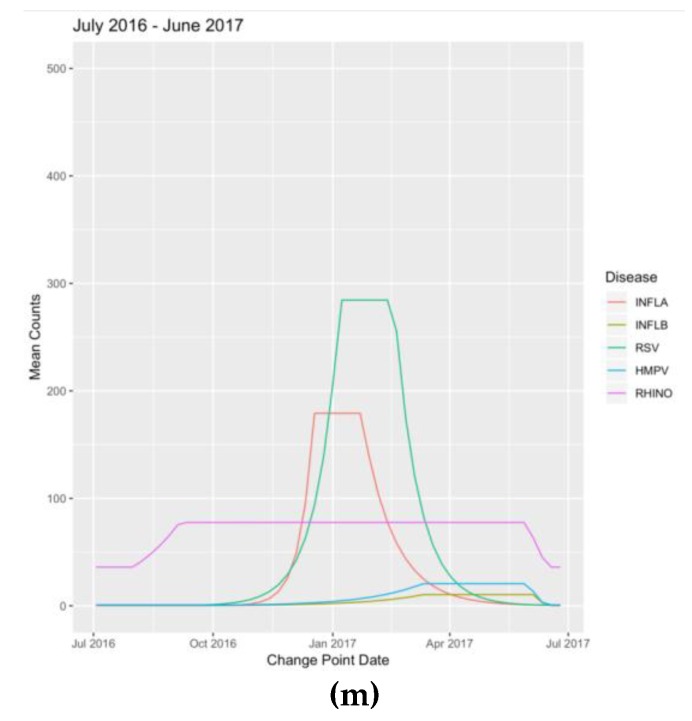
Five virus change point plots: mean count of monthly timing of positive viral samples for influenza A (INFLA), influenza B (INFLB), respiratory syncytial virus (RSV), human metapneumovirus (HMPV), and rhinovirus (RHINO) from mid-2004 (**a**) to late-2017 (**m**), in the Intermountain Health Care system. Note that November 2006 was the first month data were available for HMPV, and November 2007 was the first month data were available for RHINO, so respective viral data are provided starting after given dates. Enterovirus (EV) is plotted separately due to significant difference in mean counts and seasonal peak patterns (see Figure A2).

**Figure A2:**
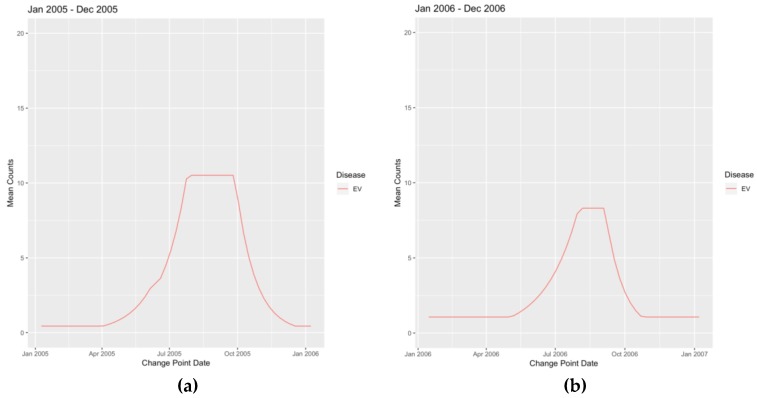

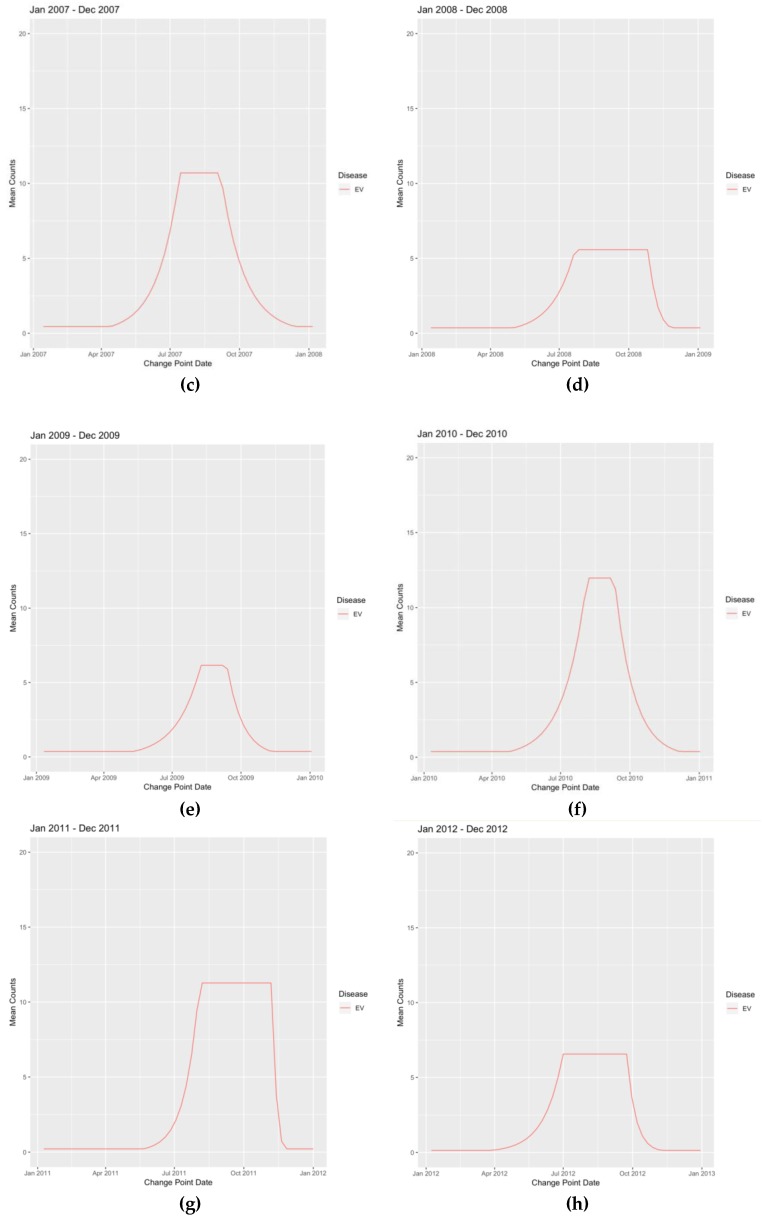

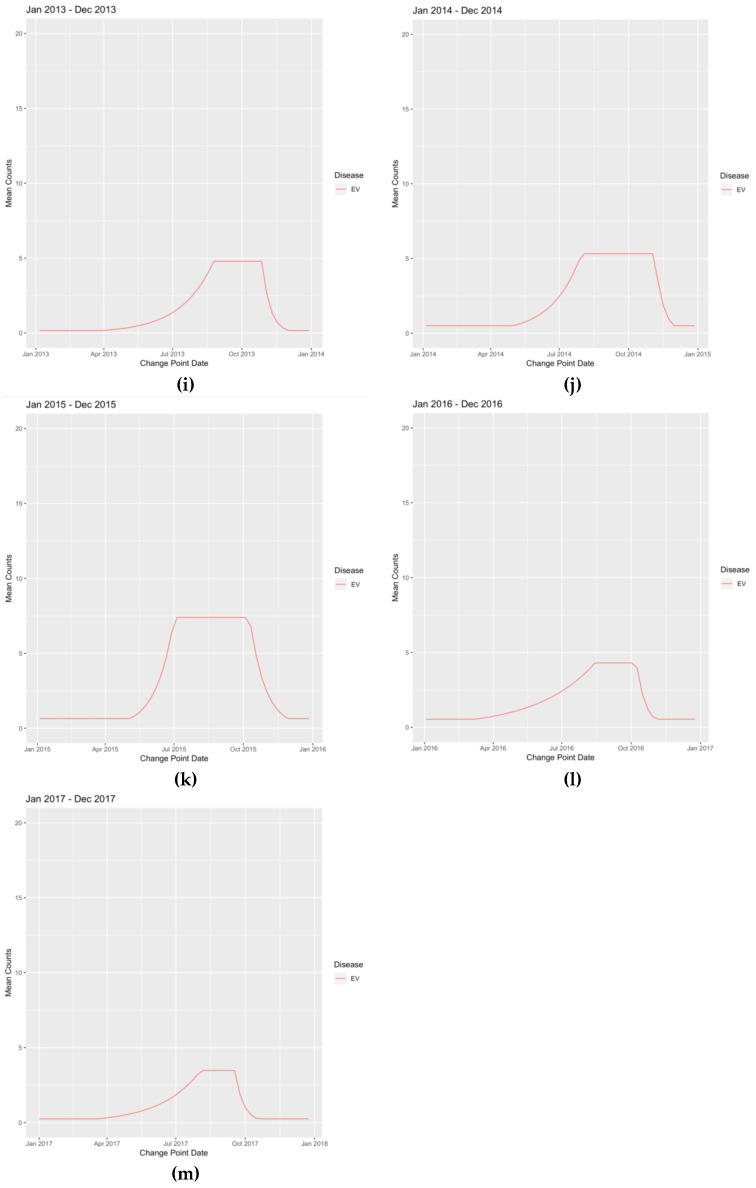
EV change point plots: for 2005 (**a**) through 2017 (**m**) Mean count of monthly timing of positive viral samples for enterovirus. Note that these charts follow a Jan-Dec seasonal cycle due to enterovirus mainly being a summer-fall peak seasonal virus.

In the change point analysis, influenza A exhibited the highest mean count in 2009 from 11 October to 18 October, with a peak mean count between 800 and 900, representative of the pandemic that year (Figure 3a). Influenza B exhibited its highest mean count in 2016, from 7 February to 28 February, with a peak mean count just above 150 (Figure 3b). RSV exhibited its highest mean count from 30 December 2012 to 17 February 2013, with a peak mean count between 307 and 362 (Figure 3c). Peak timing for influenza A overlapped eight of the 13 years with influenza B, and nine of the 13 years with RSV. (See Table A3 for all virus change point values and dates. See Figure A1 and Figure A2 for all virus change point plots.)

Enterovirus showed varied timing of peak mean frequency count and exhibited its highest mean count in 2010, from 8 October to 12 September, with a peak mean count just below 12 (Figure 3d). Metapneumovirus exhibited its highest mean count in 2010, from 14 February to 28 February, with a peak mean count between 95 and 100 (Figure 3a). Rhinovirus exhibited its highest mean count from 2 September 2012 to 12 May 2013, with a peak mean count of 111 (Figure 3c). Rhinovirus also showed the longest average yearly peak duration at a length of 27 weeks. RSV and influenza A showed the shortest average yearly peak durations at a length of five weeks (see Table A4 for all virus epidemic and peak durations).

### 3.2. Wavelet Analysis

**Figure A3:**
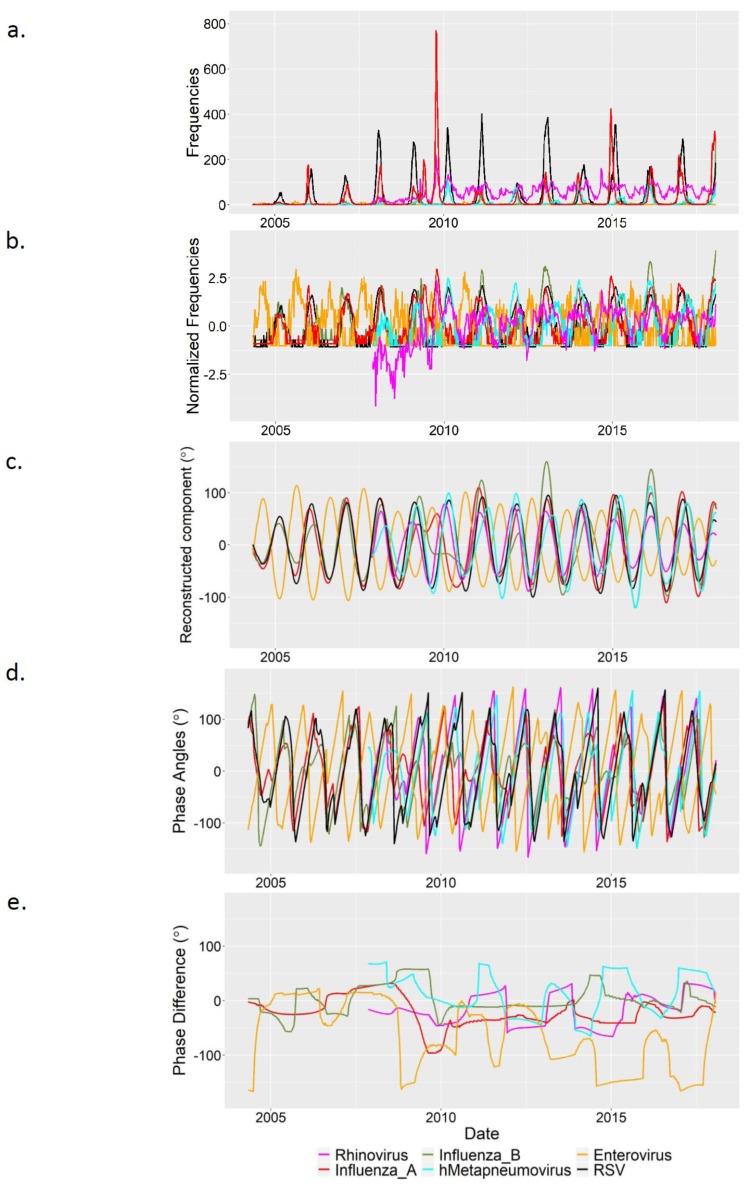
Wavelet analysis of positive viral samples from 2004 to 2018: (**a**) Raw frequency data. (**b**) Normalized frequencies. (**c**) Reconstructed component from wavelet analysis, using periods 32–65 for Rhinovirus, Metapneumovirus, RSV, and Enterovirus, and 32–108 for influenza A and B. A loess smoother was applied to rhinovirus, to correct for an increase in testing during 2007–2008. (**d**) Phase angles using the aforementioned periods. (**e**) Phase differences between each virus and RSV. Lines at zero indicate phase synchrony. Lines deviating from zero indicate the epidemics are not in phase.

**Figure A4:**
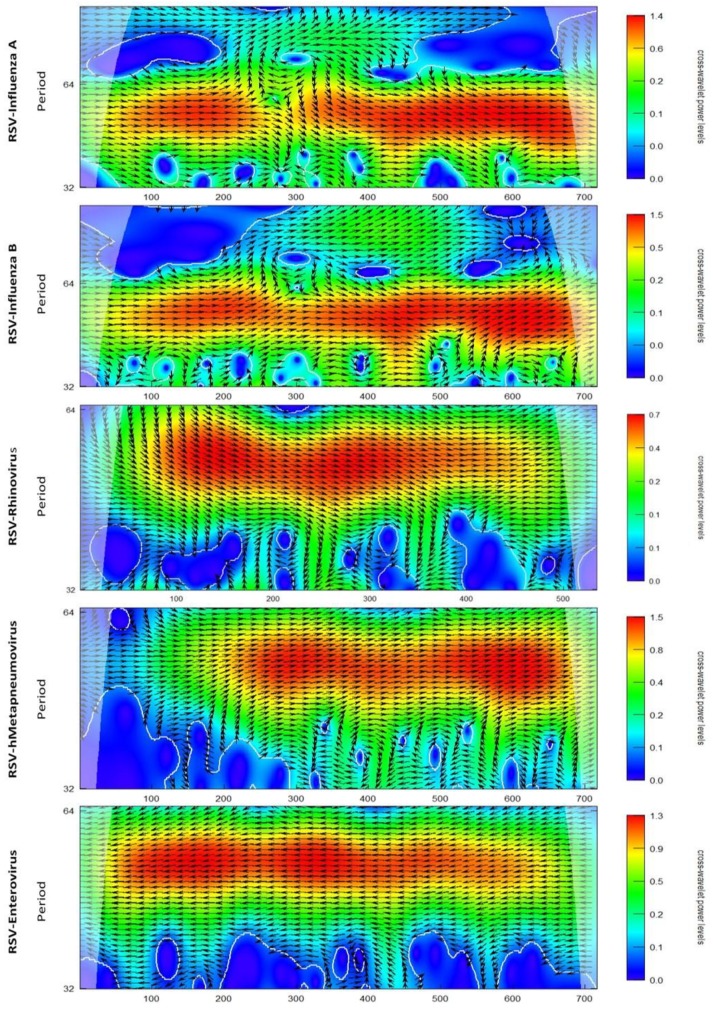
Cross-wavelet power spectrum.

Results of the wavelet analysis are shown in Figure A3, and cross-wavelet power spectrums are shown in Figure A4. Enterovirus and RSV show the least synchrony, as expected, with phase differences between approximately −50° and −180°. Influenza A and RSV show close synchrony with phase angles near 0°, except for a notable difference during the 2009–2010 H1N1 pandemic, and a shift in the 2013–2014 season. Influenza B was in synchrony with RSV, except for the 2008–2009 and 2013–2014 seasons. Metapneumovirus was out of phase with RSV in the 2011–2012 and 2013–2014 seasons, while rhinovirus moved in and out of synchrony with RSV over the study period.

## 4. Discussion

The strengths of the study include the use of a large, multiyear database of laboratory-confirmed samples and two complementary analysis methods, to determine synchrony in epidemic dynamics between six common respiratory viruses in Utah. However, interpretation of results is somewhat limited by the use of aggregate weekly frequency data rather than rates.

The first observation made from our data was visualizing the known and remarkable consistency with which RSV epidemics transmitted in Utah. The timing and duration of annual RSV epidemics were very similar across years, with peaks overlapping most years. Even in influenza pandemic years, RSV timing was not dramatically shifted.

Influenza B and metapneumovirus almost always had overlapping peaks and showed very similar trends in synchrony with RSV and overall timing. Both viruses typically peaked in February or March, but shifted to autumn during the 2010–2011 season. The wavelet analysis confirmed this phase shift, as well as showing a shift out of phase by enterovirus. The uneven pattern in enterovirus corresponds to shifts in peak timing from September to July, and then back to September. Pandemic influenza years (2009–2010) and seasons with high levels of H1N1 (2013–2014) coincided with more volatility in enterovirus and metapneumovirus synchronization.

Our results must be interpreted in light of the complex interactions that exist between viruses, populations, and the environment. Influenza A and B showed the most dynamic variability, while RSV remained markedly consistent in this population. Influenza is zoonotic, constantly experiencing genetic shift and drift, and spilling over into human populations. However, humans are the reservoir for RSV, and RSV is therefore very well adapted to human populations and may not be as driven by geographic and population-level factors as influenza. For example, temperature and humidity patterns are known to influence the spread of both RSV and influenza [20,21,22]. However, influenza epidemics are also influenced by sociodemographic characteristics of the underlying population [23,24]. It is unclear exactly how much RSV is dependent on similar population features, but previous studies support associations between respiratory viruses and human behaviors that may help explain their seasonality [25]. Previous studies showed that analysis of climate forcing and genetic drift and shift could well-predict influenza epidemic behavior [26]. Our study supports this conclusion, while also suggesting that influenza pandemic years are associated with irregularities in seasonal timing for certain other respiratory viruses. More research is needed to understand if this is due to interference by emerging influenza strains or due to some shared climate or population effect.

Rhinovirus is also well-adapted to humans but shows far less regularity in its timing than RSV. In the Utah data, rhinovirus persists at comparatively low peak levels over a much longer period of time. This is in line with other studies, some of which suggest rhinovirus may be well adapted to being present in populations at times other infections are not [27].

The delay of influenza season due to rhinovirus has been a subject of epidemiological interest for some time. For example, a particularly high-burden rhinovirus epidemic is hypothesized to have delayed the 2009 H1N1 pandemic in France [28]. Detecting interference at the population level may only be possible during more extreme events, such as pandemic influenza or higher-than-normal rhinovirus burden. Further studies with individual-level data and in different populations should be pursued to better understand why viral interference at the individual level does not necessarily translate to interference at the population level.

## Appendix A. Tables

**Table A1:** Virus yearly frequency.

Seasonal Year	Influenza A	Influenza B	RSV	HMPV	Rhinovirus	Enterovirus	Year Sums
2004	0	0	5	0	0	13	18
2005	132	67	506	0	0	124	829
2006	648	50	1181	0	0	184	2063
2007	803	219	1284	35	0	109	2450
2008	1045	212	2611	81	547	135	4631
2009	1581	240	2339	210	1419	70	5859
2010	3693	9	2359	723	3878	70	10,732
2011	717	476	2975	159	3172	125	7624
2012	391	43	1133	629	2665	64	4925
2013	924	1042	4002	250	3691	65	9974
2014	869	96	1983	821	3403	78	7250
2015	2177	218	3001	444	3962	89	9891
2016	1425	1052	1833	836	3486	107	8739
2017	1804	191	2701	378	3299	76	8449
2018	2242	852	758	379	1759	38	6028
Virus Sums	18,451	4767	28,671	4945	31,281	1347	89,462

Note: Seasonal year denotes ending year (e.g., seasonal year 2004 represents 1 July 2003 to 30 June 2004). Most of seasonal year 2004 lacks data. Seasonal year 2018 data are also incomplete. These two years were generally excluded from the analysis.

**Table A2:** Virus monthly frequency.

Month	Influenza A	Influenza B	RSV	HMPV	Rhinovirus	Enterovirus	Month Sums
January	4315	1482	8241	1190	2859	20	18,107
February	2599	1225	10,259	1258	2695	15	18,051
March	1615	736	5235	865	3363	11	11,825
April	499	333	1332	474	3096	27	5761
May	498	179	364	317	2672	70	4100
June	488	31	91	138	1585	86	2419
July	82	8	32	40	1276	274	1712
August	56	1	13	22	1748	330	2170
September	360	12	28	9	2907	231	3547
October	2500	25	88	22	3023	153	5811
November	1182	98	338	116	3065	88	4887
December	4257	637	2650	494	2992	42	11,072

**Table A3:** Virus mean count change points.

Disease	Value T1	Date T1	Value T2	Date T2	Value T3	Date T3	Value T4	Date T4
INFLA	0.13	7-Nov-2004	12.37	16-Jan-2005	12.6	13-Mar-2005	0.11	5-Jun-2005
INFLB	0.05	31-Oct-2004	14.95	9-Jan-2005	13.54	27-Feb-2005	0.06	8-May-2005
RSV	0.64	3-Oct-2004	49.42	20-Feb-2005	49.42	6-Mar-2005	0.69	19-Jun-2005
EV	0.49	8-Aug-2004	11.13	14-Nov-2004	11.13	16-Jan-2005	0.44	10-Apr-2005
HMPV	NA	NA	NA	NA	NA	NA	NA	NA
RHINO	NA	NA	NA	NA	NA	NA	NA	NA
INFLA	0.39	6-Nov-2005	189.32	18-Dec-2005	189.32	25-Dec-2005	0.52	26-Feb-2006
INFLB	0.12	2-Oct-2005	7.38	5-Feb-2006	7.47	16-Apr-2006	0.12	28-May-2006
RSV	0.23	2-Oct-2005	147.08	8-Jan-2006	117.85	12-Feb-2006	0.28	14-May-2006
EV	1.08	21-Aug-2005	7.5	4-Dec-2005	7.38	1-Jan-2006	1.1	19-Feb-2006
HMPV	NA	NA	NA	NA	NA	NA	NA	NA
RHINO	NA	NA	NA	NA	NA	NA	NA	NA
INFLA	0.36	3-Sep-2006	76.25	11-Feb-2007	81.69	4-Mar-2007	0.36	24-Jun-2007
INFLB	0.12	15-Oct-2006	16.36	10-Dec-2006	17.15	18-Feb-2007	0.12	13-May-2007
RSV	0.39	15-Oct-2006	110.52	14-Jan-2007	115.63	4-Mar-2007	0.37	24-Jun-2007
EV	0.49	13-Aug-2006	10.08	12-Nov-2006	9.28	7-Jan-2007	0.45	15-Apr-2007
HMPV	0.05	26-Nov-2006	18.53	31-Dec-2006	13.65	4-Mar-2007	0.04	15-Apr-2007
RHINO	NA	NA	NA	NA	NA	NA	NA	NA
INFLA	0.76	18-Nov-2007	178.58	10-Feb-2008	178.58	24-Feb-2008	0.97	4-May-2008
INFLB	0.17	2-Dec-2007	28.62	10-Feb-2008	27.92	16-Mar-2008	0.17	11-May-2008
RSV	0.48	7-Oct-2007	326.24	20-Jan-2008	268.18	17-Feb-2008	0.49	8-Jun-2008
EV	0.36	26-Aug-2007	3.94	18-Nov-2007	4.24	24-Feb-2008	0.44	23-Mar-2008
HMPV	0.1	25-Nov-2007	78.52	27-Jan-2008	70.01	1-Jun-2008	0.07	22-Jun-2008
RHINO	0.72	4-Nov-2007	65.33	25-Nov-2007	45.06	15-Jun-2008	1.3	29-Jun-2008
INFLA	2.8	28-Dec-2008	112.59	1-Feb-2009	53.08	28-Jun-2009	53.08	28-Jun-2009
INFLB	0.19	21-Dec-2008	62.63	22-Mar-2009	46.99	10-May-2009	0.2	21-Jun-2009
RSV	0.38	19-Oct-2008	263.44	1-Feb-2009	263.44	1-Mar-2009	0.38	28-Jun-2009
EV	0.37	7-Sep-2008	8.01	7-Dec-2008	7.63	11-Jan-2009	0.41	8-Mar-2009
HMPV	0.28	30-Nov-2008	22.05	15-Feb-2009	22.05	26-Apr-2009	0.35	31-May-2009
RHINO	20.27	25-Jan-2009	47.89	26-Apr-2009	27.22	28-Jun-2009	27.22	28-Jun-2009
INFLA	2.52	9-Aug-2009	803.25	11-Oct-2009	917.61	18-Oct-2009	2.52	3-Jan-2010
INFLB	0.03	23-Aug-2009	3.65	27-Sep-2009	3.65	18-Oct-2009	0.04	29-Nov-2009
RSV	0.77	8-Nov-2009	337.97	7-Feb-2010	336.19	28-Feb-2010	0.76	20-Jun-2010
EV	0.39	16-Aug-2009	11.33	6-Dec-2009	10.92	10-Jan-2010	0.39	4-Apr-2010
HMPV	0.28	25-Oct-2009	100.25	14-Feb-2010	95.22	28-Feb-2010	0.28	13-Jun-2010
RHINO	24.82	16-Aug-2009	106.66	4-Oct-2009	106.66	28-Mar-2010	25.04	27-Jun-2010
INFLA	0.16	29-Aug-2010	54.28	19-Dec-2010	54.28	13-Feb-2011	0.19	15-May-2011
INFLB	0.13	24-Oct-2010	82.83	13-Feb-2011	65.51	27-Feb-2011	0.12	22-May-2011
RSV	0.81	24-Oct-2010	359.79	6-Feb-2011	298.5	6-Mar-2011	0.74	26-Jun-2011
EV	0.2	26-Sep-2010	17.22	28-Nov-2010	9.02	13-Mar-2011	0.2	27-Mar-2011
HMPV	0.25	14-Nov-2010	10.47	24-Apr-2011	11.18	26-Jun-2011	11.18	26-Jun-2011
RHINO	34.28	18-Jul-2010	71.96	31-Oct-2010	68.09	22-May-2011	35.56	5-Jun-2011
INFLA	0.27	11-Dec-2011	64.15	4-Mar-2012	68.63	18-Mar-2012	0.33	10-Jun-2012
INFLB	0.11	11-Dec-2011	4.32	26-Feb-2012	1.87	3-Jun-2012	0.11	17-Jun-2012
RSV	1.59	6-Nov-2011	77.52	19-Feb-2012	80.14	8-Apr-2012	1.59	1-Jul-2012
EV	0.14	24-Jul-2011	6.02	30-Oct-2011	6.09	22-Jan-2012	0.18	4-Mar-2012
HMPV	0.53	23-Oct-2011	71.79	5-Feb-2012	75.82	26-Feb-2012	0.5	27-May-2012
RHINO	28.29	24-Jul-2011	65.47	13-Nov-2011	65.94	29-Apr-2012	30.5	13-May-2012
INFLA	0.44	14-Oct-2012	188.14	30-Dec-2012	188.14	20-Jan-2013	0.39	21-Apr-2013
INFLB	0.19	16-Sep-2012	89.48	16-Dec-2012	82.1	3-Feb-2013	0.2	9-Jun-2013
RSV	0.58	16-Sep-2012	361.83	30-Dec-2012	307.44	17-Feb-2013	0.66	16-Jun-2013
EV	0.17	29-Jul-2012	4.79	23-Dec-2012	4.81	24-Feb-2013	0.18	31-Mar-2013
HMPV	0.51	23-Sep-2012	14.82	28-Apr-2013	2.43	23-Jun-2013	2.43	23-Jun-2013
RHINO	37.46	5-Aug-2012	111.59	2-Sep-2012	111.59	12-May-2013	36.56	16-Jun-2013
INFLA	0.47	6-Oct-2013	139.96	22-Dec-2013	139.66	29-Dec-2013	0.41	18-May-2014
INFLB	0.5	15-Dec-2013	9.83	13-Apr-2014	10.06	11-May-2014	0.55	8-Jun-2014
RSV	1.51	13-Oct-2013	147.94	2-Feb-2014	147.94	23-Mar-2014	1.75	22-Jun-2014
EV	0.51	1-Sep-2013	5.12	24-Nov-2013	5.45	2-Mar-2014	0.53	30-Mar-2014
HMPV	1.71	29-Sep-2013	83.57	29-Dec-2013	82.58	12-Jan-2014	1.55	18-May-2014
RHINO	32.91	18-Aug-2013	77.13	8-Sep-2013	80.62	18-May-2014	34.15	15-Jun-2014
INFLA	0.24	12-Oct-2014	359.06	14-Dec-2014	425.62	21-Dec-2014	0.21	31-May-2015
INFLB	0.22	7-Sep-2014	14.18	22-Mar-2015	10.81	17-May-2015	0.35	7-Jun-2015
RSV	1.05	21-Sep-2014	318.54	25-Jan-2015	343.49	15-Feb-2015	1.12	31-May-2015
EV	0.7	31-Aug-2014	6.91	2-Nov-2014	6.36	8-Feb-2015	0.65	29-Mar-2015
HMPV	0.28	26-Oct-2014	27.03	8-Mar-2015	14.98	7-Jun-2015	0.46	21-Jun-2015
RHINO	42.48	10-Aug-2014	82.43	31-Aug-2014	91.02	24-May-2015	39.21	21-Jun-2015
INFLA	0.51	1-Nov-2015	157.73	14-Feb-2016	159.17	20-Mar-2016	0.52	12-Jun-2016
INFLB	0.53	15-Nov-2015	152.9	7-Feb-2016	150.72	28-Feb-2016	0.44	12-Jun-2016
RSV	1.07	27-Sep-2015	143.45	24-Jan-2016	149.23	6-Mar-2016	0.98	19-Jun-2016
EV	0.56	5-Jul-2015	4.16	20-Dec-2015	3.85	7-Feb-2016	0.67	28-Feb-2016
HMPV	0.81	11-Oct-2015	80.3	24-Jan-2016	68.35	28-Feb-2016	0.81	29-May-2016
RHINO	55.46	27-Dec-2015	97.15	6-Mar-2016	99.8	8-May-2016	54.9	5-Jun-2016
INFLA	0.91	23-Oct-2016	179.9	18-Dec-2016	179.9	22-Jan-2017	1.03	28-May-2017
INFLB	0.23	25-Sep-2016	10.18	5-Mar-2017	3.34	11-Jun-2017	0.24	18-Jun-2017
RSV	0.74	18-Sep-2016	262.02	8-Jan-2017	234.19	19-Feb-2017	0.62	11-Jun-2017
EV	0.26	17-Jul-2016	3.36	4-Dec-2016	3.32	15-Jan-2017	0.31	12-Feb-2017
HMPV	0.46	25-Sep-2016	19.91	12-Mar-2017	13.04	4-Jun-2017	0.8	18-Jun-2017
RHINO	37.25	31-Jul-2016	69.49	4-Sep-2016	72.69	28-May-2017	35.87	18-Jun-2017

Note: T1–T4 indicate change points in the virus cycle. Values indicate weekly mean count. T1 indicates virus epidemic onset. T2 indicates end of onset and start of peak. T3 represents end of peak and start of offset. T4 represents end of offset.

**Table A4:** Virus peak and epidemic durations.

Seasonal Year	Duration (Weeks)	Influenza A	Influenza B	RSV	HMPV	Rhinovirus	Enterovirus
2004–2005	Peak	8	7	2	9	NA	NA
	Epidemic	30	27	37	35	NA	NA
2005–2006	Peak	1	10	5	4	NA	NA
	Epidemic	16	34	32	26	NA	NA
2006–2007	Peak	3	10	7	8	9	NA
	Epidemic	42	30	36	35	20	NA
2007–2008	Peak	2	5	4	14	18	29
	Epidemic	24	23	35	30	30	34
2008–2009	Peak	21	7	4	5	10	9
	Epidemic	26	26	36	26	26	22
2009–2010	Peak	1	3	3	5	2	25
	Epidemic	21	14	32	33	33	45
2010–2011	Peak	8	2	4	15	9	29
	Epidemic	37	30	35	26	32	46
2011–2012	Peak	2	14	7	12	3	24
	Epidemic	26	27	34	32	31	42
2012–2013	Peak	3	7	7	9	8	36
	Epidemic	27	38	39	35	39	45
2013–2014	Peak	1	4	7	14	2	36
	Epidemic	32	25	36	30	33	43
2014–2015	Peak	1	8	3	14	13	38
	Epidemic	33	39	36	30	34	45
2015–2016	Peak	5	3	6	7	5	9
	Epidemic	32	30	38	34	33	23
2016–2017	Peak	5	14	6	6	12	38
	Epidemic	31	38	38	30	38	46

Note: Peak duration reflects length of time virus maintains highest mean frequency count, measured as weeks between T2 and T3. Epidemic duration reflects length of time virus is generally in circulation, measured as weeks between T1 and T4.

## References

[1] Nair H., Nokes D.J., Gessner B.D., Dherani M., Madhi S.A., Singleton R.J., O’Brien K.L., Roca A., Wright P.F., Bruce N. (2010). Global burden of acute lower respiratory infections due to respiratory syncytial virus in young children: A systematic review and meta-analysis. Lancet.

[2] Dawood F.S., Iuliano A.D., Reed C., Meltzer M.I., Shay D.K., Cheng P.-Y., Bandaranayake D., Breiman R.F., Brooks W.A., Buchy P. (2012). Estimated global mortality associated with the first 12 months of 2009 pandemic influenza A H1N1 virus circulation: A modelling study. Lancet Infect. Dis..

[3] Rose E.B., Wheatley A., Langley G., Gerber S., Haynes A. (2018). Respiratory Syncytial Virus Seasonality-United States, 2014–2017. Mmwr. Morb. Mortal. Wkly. Rep..

[4] Centers for Disease Control and Prevention Overview of Influenza Surveillance in the United States. https://www.cdc.gov/flu/weekly/overview.htm.

[5] Centers for Disease Control and Prevention (2018). Respiratory Syncytial Virus Infection (RSV): Trends and Surveillance. https://www.cdc.gov/rsv/research/us-surveillance.html.

[6] Esper F.P., Spahlinger T., Zhou L. (2011). Rate and influence of respiratory virus co-infection on pandemic (H1N1) influenza disease. J. Infect..

[7] Debiaggi M., Canducci F., Ceresola E.R., Clementi M. (2012). The role of infections and coinfections with newly identified and emerging respiratory viruses in children. Virol. J..

[8] Lam T.T., Tang J.W., Lai F.Y., Zaraket H., Dbaibo G., Bialasiewicz S., Tozer S., Heraud J.M., Drews S.J., Hachette T. (2019). Comparative global epidemiology of influenza, respiratory syncytial and parainfluenza viruses, 2010–2015. J. Infect..

[9] Pinky L., Dobrovolny H.M. (2016). Coinfections of the Respiratory Tract: Viral Competition for Resources. PLoS ONE.

[10] Laurie K.L., Guarnaccia T.A., Carolan L.A., Yan A.W.C., Aban M., Petrie S., Cao P., Heffernan J.M., McVernon J., Mosse J. (2015). Interval Between Infections and Viral Hierarchy Are Determinants of Viral Interference Following Influenza Virus Infection in a Ferret Model. J. Infect. Dis..

[11] Van Asten L., Bijkerk P., Fanoy E., van Ginkel A., Suijkerbuijk A., van der Hoek W., Meijer A., Vennema H. (2016). Early occurrence of influenza A epidemics coincided with changes in occurrence of other respiratory virus infections. Influenza Other Respir. Viruses.

[12] Karppinen S., Toivonen L., Schuez-Havupalo L., Waris M., Peltola V. (2016). Interference between respiratory syncytial virus and rhinovirus in respiratory tract infections in children. Clin. Microbiol. Infect..

[13] Viboud C., Bjørnstad O.N., Smith D.L., Simonsen L., Miller M.A., Grenfell B.T. (2006). Synchrony, Waves, and Spatial Hierarchies in the Spread of Influenza. Science.

[14] Grenfell B.T., Bjørnstad O.N., Kappey J. (2001). Travelling waves and spatial hierarchies in measles epidemics. Nature.

[15] R Core Team (2018). R: A Language and Environment for Statistical Computing.

[16] Pugh S., Heaton M.J., Hartman B., Berrett C., Sloan C., Evans A.M., Gebretsadik T., Wu P., Hartert T.V., Lee R.L. (2019). Estimating seasonal onsets and peaks of bronchiolitis with spatially and temporally uncertain data. Stat. Med..

[17] Centers for Disease Control and Prevention (2019). The National Respiratory and Enteric Virus Surveillance System (NREVSS): RSV Surveillance Reports. https://www.cdc.gov/surveillance/nrevss/rsv/reports.html.

[18] Roesch A., Schmidbauer H. WaveletComp: Computational Wavelet Analysis; R package version 1.1; 2018. https://www.researchgate.net/publication/323836523.

[19] Almeida A., Codeço C., Luz P.M. (2018). Seasonal dynamics of influenza in Brazil: The latitude effect. BMC Infect. Dis..

[20] Lowen A.C., Steel J. (2014). Roles of humidity and temperature in shaping influenza seasonality. J. Virol..

[21] Lowen A.C., Steel J., Mubareka S., Palese P. (2008). High temperature (30 C) blocks aerosol but not contact transmission of influenza virus. J. Virol..

[22] Yunus A.S., Jackson T.P., Crisafi K., Burimski I., Kilgore N.R., Zoumplis D., Allaway G.P., Wild C.T., Salzwedel K. (2010). Elevated temperature triggers human respiratory syncytial virus F protein six-helix bundle formation. Virology.

[23] Sloan C., Chandrasekhar R., Mitchel E., Schaffner W., Lindegren M.L. (2015). Socioeconomic Disparities and Influenza Hospitalizations, Tennessee, USA. Emerg. Infect. Dis..

[24] Yang L., Hung Chan K., Suen L.K.P., Pan Chan K., Wang X., Cao P., He D., Malik Peiris J.S., Ming Wong C. (2015). Age-specific epidemic waves of influenza and respiratory syncytial virus in a subtropical city. Sci. Rep..

[25] Sloan C., Moore M.L., Hartert T. (2011). Impact of Pollution, Climate, and Sociodemographic Factors on Spatiotemporal Dynamics of Seasonal Respiratory Viruses. Clin. Transl. Sci..

[26] Axelsen J.B., Yaari R., Grenfell B.T., Stone L. (2014). Multiannual forecasting of seasonal influenza dynamics reveals climatic and evolutionary drivers. Proc. Natl. Acad. Sci. USA.

[27] Monto A.S. (2002). The seasonality of rhinovirus infections and its implications for clinical recognition. Clin. Ther..

[28] Casalegno J.S., Ottmann M., Bouscambert Duchamp M., Escuret V., Billaud G., Frobert E., Morfin F., Lina B. (2010). Rhinoviruses delayed the circulation of the pandemic influenza A (H1N1) 2009 virus in France. Clin. Microbiol. Infect..

